# Is Exergaming a Viable Tool in the Fight against Childhood Obesity?

**DOI:** 10.1155/2014/304521

**Published:** 2014-03-11

**Authors:** Gary S. Goldfield, Jameason D. Cameron, Jean-Philippe Chaput

**Affiliations:** Healthy Active Living and Obesity Research Group, Children's Hospital of Eastern Ontario Research Institute, Ottawa, ON, Canada K1H 8L1

In a recent issue of this journal, Lamboglia and colleagues published a review paper entitled “Exergaming as a strategic tool in the fight against childhood obesity: a systematic review” [1]. The rationale for this review was that physical activity levels among children and adolescents are very low, and time spent in sedentary behaviors, especially screen time, is very high in Westernized countries, and this is problematic given that both are known risk factors for childhood obesity and associated comorbidity. Thus, a systematic review of exergaming (also known as active video gaming) was conducted as it may represent an enjoyable way in which children can capitalize on advances in technology to increase energy expenditure and cardiovascular fitness and improve body composition to “combat childhood obesity.”

This review included 9 studies published from January 2008 to April 2012. Six of the 9 studies included were acute laboratory studies, with 3 intervention studies ranging from 12 to 28 weeks. The results indicate that exergaming increased physical activity levels, energy expenditure, maximal oxygen uptake, and heart rate and reduced sedentary screen time and waist circumference. As such, the authors concluded that “exergaming can be considered a highly relevant strategic tool for the adoption of an active and healthy lifestyle and may be useful in the fight against childhood obesity.” However, we believe that such a conclusion is premature and not supported by the current state of knowledge on this topic.

As the authors note, the systematic review included a very small number of studies, and the majority of studies (6 of the 9) were conducted in the laboratory. In the three randomized controlled intervention studies reviewed, all of which took place in the home environment, the effects of exergaming on body composition were mixed. One study found no effect on BMI [2], another showed no effect on BMI but a reduction in waist circumference [3], and a large study found small but statistically significant reductions in BMI and percent body fat [4]. Moreover, differences between intervention and control groups on volume and intensity of physical activity and time spent in seated video games were not consistently found. Given these inconsistent findings, the authors' conclusions seem to be based on the more consistent findings of increased energy expenditure of exergaming in comparison to seated video games in the laboratory studies.

While the data from this review clearly support increased energy expenditure from exergaming in laboratory studies (relative to sitting), it is important to note these are acute effects, and since food intake was not measured in any of these studies, it is impossible to determine the net influence of exergaming on energy balance. More specifically, increases in energy expenditure from exergaming may have little impact on child obesity prevention or treatment if children compensate by increasing energy intake, which the research suggests is a common phenomenon [5]. The coupling of high energy expenditure with compensatory increases in food intake may explain why meta-analytic reviews show that exercise alone in the absence of dietary changes is ineffective in the treatment of childhood obesity [6]. Moreover, leisure time physical activity throughout the day was not measured in the laboratory studies noted by Lamboglia et al. [1]. This is a significant limitation given the research showing that acute increases in energy expenditure can be followed by compensatory decreases in leisure time physical activity in children [7, 8]. This concept is known as “activitystat” and this regulating mechanism may further explain why physical activity alone rarely leads to sustained states of negative energy balance needed for weight loss. Furthermore, a recent and more comprehensive systematic review by Active Healthy Kids Canada indicates that the acute increases in energy expenditure induced by exergaming are not sustained over time, thereby limiting the health benefits and minimizing the impact on childhood obesity [9, 10]. Taken together, we argue that the heterogeneity of the methodologies used between experiments reported in the review article by Lamboglia et al. [1] is the main limitation to the authors' premature conclusions. In order to make population-based recommendations regarding the strategic benefits of exergaming there is a need to perform a more rigorous assessment of energy expenditure and a more precise account of energy intake, both in the laboratory and in the natural environment. In combination with such sophisticated measures of bioenergetics, it will also be imperative to disentangle the effects of frequency, intensity, and duration of these exergames, along with isolating the impact of more chronic interventions.

In conclusion, the evidence from this review supports the use of exergaming to acutely increase energy expenditure compared to seated video games. Considering that seated video games increase food intake [11], exergaming should be encouraged to replace seated video games and may be helpful in achieving recommended physical activity levels in children worldwide. However, given the mixed evidence of impact on physical activity and body composition in the intervention studies, combined with research showing that acute increases in energy expenditure can be followed by compensatory adjustments in food intake and/or leisure time energy expenditure, exergaming is unlikely to be an effective tool to combat childhood obesity. While further intervention studies of exergaming are clearly warranted (especially under naturalistic conditions), it is paramount that future research focuses on evaluating innovative but comprehensive solutions that address the complex, multifactorial etiology of childhood obesity. In the meantime, physical activity in the natural environment with associated benefits of fresh air, vitamin D, connection with nature, and meaningful social interactions should be promoted over exergaming.
